# Fracture Resistance of Monolithic High-Translucency Crowns Versus Porcelain-Veneered Zirconia Crowns After Artificial Aging: An In Vitro Study

**DOI:** 10.7759/cureus.20640

**Published:** 2021-12-23

**Authors:** Yousef Ezzat, Rayan Sharka, Mohammad Rayyan, Mohammed Al-Rafee

**Affiliations:** 1 Dentistry, College of Dentistry, Riyadh Elm University, Riyadh, SAU; 2 Dentistry, College of Dentistry, Umm Al-Qura University, Makkah, SAU

**Keywords:** high-translucency monolithic zirconia (htz), porcelain-veneered zirconia (pvz), computer-aided design/computer-assisted manufacture (cad/cam), fixed prosthodontics, zirconia, fracture resistance, digital dentistry

## Abstract

Purpose

To evaluate the fracture resistance and fracture mode of high-translucency monolithic zirconia (HTZ) crowns and porcelain-veneered zirconia (PVZ) crowns.

Material and methods

A master die was scanned to design and fabricate the HTZ group (n = 10) and PVZ group (n = 10). Both groups were artificially aged before loaded to fracture. The means of fracture loads of the two groups were compared using an independent t-test at a significance level of 0.05. The mode of fracture was determined using a digital magnifier.

Results

The mean fracture strength for the HTZ group (4,425 ± 177 newtons (N)) was significantly higher than in the PVZ group (1,798 ± 30.9 N) (p-value < 0.001). All crowns in the HTZ group presented core fracture mode. However, crowns in the PVZ group showed both a core and adhesive fracture mode of 60% and 40%, respectively.

Conclusion

The fracture strength of HTZ crowns is superior to PVZ crowns. The fracture strength of both types surpassed the maximum bite force in the posterior region, which may be deemed clinically adequate.

## Introduction

The need for ceramic restorations has risen over the past years. As an outcome, a substantial number of studies have been conducted to examine the reliability of ceramic restorations for application in posterior restorations. Yttrium oxide-stabilized zirconia is considered the strongest tooth-coloured restorative material available nowadays. Its superior flexural strength of 900 to 1,200 megapascals (MPa) and excellent biocompatibility make it a favourite option among other all-ceramic systems. However, due to the inherent opaque nature of zirconia, it is often used as a coping and veneered with a layer of a more translucent ceramic-like feldspathic porcelain [1].

Yttria-tetragonal zirconia polycrystal (Y-TZP) coping veneered with feldspathic porcelain presented superior mechanical properties as well as excellent aesthetics [2]. Although this material showed promising results, the failure reported frequently is the chipping of the veneering porcelain which has been related to different factors, including the design and surface treatment of zirconia core and the mismatch of coefficients of thermal expansion between the core and the veneering layer [3-9]. 

Recently, computer-aided design/computer-assisted manufacture (CAD/CAM) has significantly improved all-ceramic restorations in terms of the varieties of material that can be utilized, the better ways that dies are handled and produced, and how crowns can be manufactured without a master die model [10].

By employing a CAD/CAM system, the zirconia core and veneer layers are designed mutually in the CAD software. After a standard zirconia core fabrication process, the veneer layer will be milled from lithium disilicate glass-ceramic with consolidation of the core and veneer employing a glass-ceramic powder. This process has helped to decrease the working time, improve cost-effectiveness, and optimize the control of shade and design of zirconia restorations [11].

Another way to improve the strength of zirconia restorations is to use monolithic restorations [12-14]. A full crown can be milled using one block of material which reduces complications, such as porcelain chipping and delamination. Also, monolithic zirconia crowns claim to show better mechanical properties than porcelain-veneered zirconia restorations. However, scarce studies have examined the fracture resistance of monolithic zirconia crowns compared to porcelain-veneered zirconia crowns in posterior teeth.

Therefore, the current study aims to compare the fracture resistance of posterior high-translucency monolithic zirconia (HTZ) crowns and porcelain-veneered zirconia (PVZ) crowns. Also, we will examine the fracture mode of both types of crowns. The null hypothesis is that there is no significant difference in the mean fracture resistance between the PVZ and HTZ groups.

## Materials and methods

Fabrication of the PVZ group

To manufacture zirconia copings for the PVZ group, an artificial maxillary right first molar tooth (CEREC AC Model) (Ivoclar Vivadent AG, Schaan, Liechtenstein) was prepared to receive an all-ceramic crown. The preparation had a 1 mm heavy chamfer, a 2 mm occlusal reduction, and a total convergence angle of 10 degrees. The demo model was scanned using the Smart Optical 3D laser scanner (Open Technologies, Rezzato, Italy). The data were then uploaded into the Exocad software (Exocad GmbH, Darmstadt, Germany) to mill 20 metal dies (Metal Alloy, Mesa Italia SRL, Brescia, Italy) in a milling machine (Roland Corp., Los Angeles, CA, USA) [15].

The metal dies were scanned using the Smart Optical 3D intraoral scanner to design 10 zirconia copings by using Exocad software with parameters set at 0.6 mm thickness and a 30 μm spacer.

**Figure 1:**
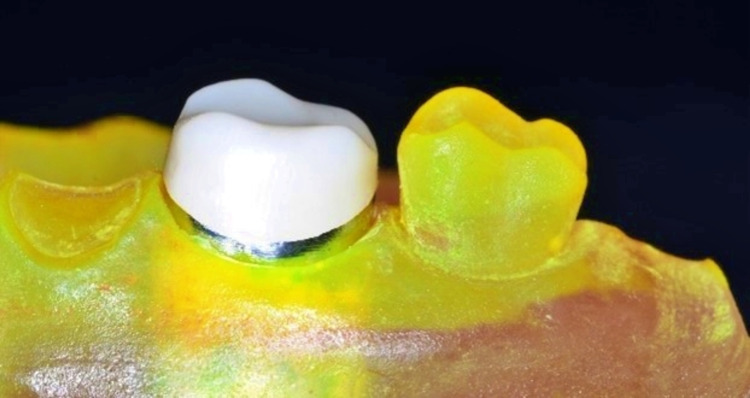
Zirconia coping on the metal die

The 10 copings were milled using pre-sintered Y-TZP materials (Zircostar®, Kerox Dental, Sóskút, Hungary) and the sintering procedure was followed to the manufacturer's specifications (Figure 1).

The porcelain veneer layer was designed by using the Exocad software with 0.7 mm thickness cervically and 1.5 mm at occlusal surfaces. The millable wax was used to connect the zirconium oxide frameworks to the castable wax. Each wax veneer was sprued and invested by IPS PressVEST Speed investment material and the formed mold was filled with the pressable glass-ceramic ingots (IPS e.max ZirPress Ingots) (Ivoclar Vivadent AG, Schaan, Liechtenstein). Each crown underwent a firing cycle in a ceramic furnace (Programat EP 3010; Ivoclar Vivadent AG, Schaan, Liechtenstein) following the manufacturer's instruction [15]. 

All crowns were prepared with finishing burs and silicon carbide paper (1400 and 2000 grit, 401Q) (3M, St. Paul, MN, USA), then coated with IPS e.max Ceram Glaze (Ivoclar Vivadent AG, Schaan, Liechtenstein) as per the manufacturer's instructions.

Fabrication of HTZ group

**Figure 2:**
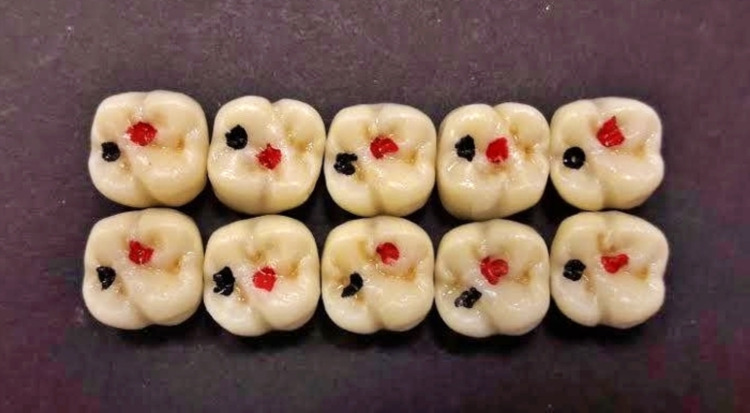
Monolithic high-translucency Y-TZP crowns (HTZ group) HTZ: high-translucency monolithic zirconia; Y-TZP: yttria-tetragonal zirconia polycrystal

Similarly, the metal dies were scanned to design and mill 10 fully anatomical high-translucency crowns for the HTZ group from a high- translucency zirconia blank (ZircoStar). One of the crowns from the PVZ group was scanned by the Smart Optical 3D scanner and used as a bio-copy to design the translucent monolithic zirconia crowns with a 30 μm spacer using Exocad software. The crowns were sintered in a furnace at 1,450° C to attain their full strength and glazed using Vita AKZENT Plus (VITA Zahnfabrik, Bad Säckingen, Germany) at glazing temperatures specified by manufacturer's instructions (Figure 2).

The first stage of artificial aging

Two stages of artificial aging were applied: thermocycling and cyclic preload. In the thermocycling stage, all 20 crowns underwent 3,000 cycles in two water baths at temperatures of 5° C and 55° C in a thermocycler machine (THE‑1100‑SD) (Mechatronik GmbH, Feldkirchen-Westerham, Germany). Every cycle persisted for 60 seconds; 20 seconds in each bath and 10 seconds for the change between the baths.

Cementation 

Using a dental surveyor, the metal dies were installed vertically in a self-cured orthodontic acrylic resin. The size of the holder in the masticatory machine used for the preloading experiments dictated the size of the cylindrical silicone mold, which was 35 mm in diameter. According to the manufacturer's directions, the 20 crowns were bonded to the corresponding metal die with glass-ionomer cement (Ketac™ Cem Aplicap™, 3M ESPE, Seefeld, Germany), and a uniform seating load of 15 newtons (N) was applied in the direction of insertion for 60 seconds [15].

After cementation, the crowns were stored in a plastic container with water covering the bottom surface and a sealed lid to create a humid atmosphere resembling the oral cavity to avoid desiccation of the luting cement.

The second stage of artificial aging

In this subsequent stage of artificial aging, the cyclic was preloaded. Also, a three-point contact between the occlusal surface of each crown and the indenter was confirmed on cusp slopes. Each crown was cyclically pre-loaded in wet conditions for 10,000 cycles using a using chewing stimulating (chewing simulator CS-4) machine (SD Mechatronik GmbH, Feldkirchen-Westerham Germany). The loads were between 30 and 300 N with a load frequency of 1 Hz that was parallel to the vertical plane.

Load to fracture

After being stored in distilled water for 24 hours, the crowns were mounted in a vertical position; the load was applied toward the occlusal surface using a 3.7 mm diameter stainless steel ball and a 0.5 mm/min crosshead speed using an Instron® 5960 universal testing machine (Instron®, High Wycombe, UK). The fracture was described as the occurrence of visible cracks in combination with load drops and acoustic events.

Analysis of fracture mode

Inspection of the broken crowns and fractured fragments was conducted, and the fracture mode (core fractures, adhesive or cohesive fractures) was ascertained by using a Mobiloskop magnifier (Renfert GmbH, Hilzingen, Germany). 

Statistical analysis

The sample of 10 crowns for each group was chosen based on previous in vitro studies [5-6]. It achieves 85% power to detect differences among the means with a .05 (α) significance level based on (G*power v 3.1) statistical software [16].

The statistical analysis was performed by using IBM Statistical Product and Service Solutions (SPSS), version 25 (IBM SPSS Statistics for Windows, Armonk, NY). The means of fracture loads of the two groups were compared using an independent t-test. A p-value below 0.05 was considered statistically significant.

## Results

Fracture load

**Figure 3:**
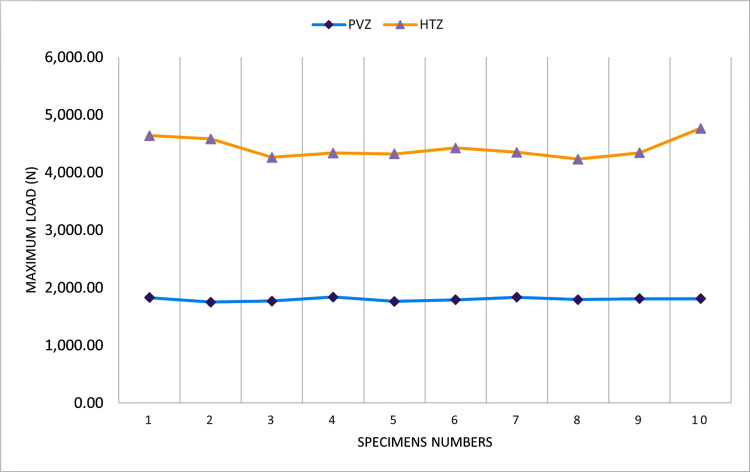
Fracture resistance loads in the samples from both HTZ and PVZ groups HTZ: high-translucency monolithic zirconia; N: newtons; PVZ: porcelain-veneered zirconia

Table 1 presents the means, maximum and minimum values, and standard deviations (SDs) of the measured fracture loads. Independent t-test showed significant differences in fracture loads between the two groups. The HTZ group showed higher fracture loads (mean = 4,425 N) compared to the PVZ group (mean = 1,798 N) (p-value < 0.001). The overall mean fracture resistance loads in each specimen of both groups are presented in Figure 3.

**Table 1:** Means, Maximum and Minimum Values, and Standard Deviations (SDs) of the Measured Fracture Loads HTZ: high-translucency monolithic zirconia; PVZ: porcelain-veneered zirconia

Group	Mean	Maximum	Minimum	SDs	P-value
HTZ	4,425.0	4,765.9	4,229.4	177.2	< 0.001
PVZ	1,797.5	1,837.2	1,748.8	30.9	

Fracture mode

**Figure 4:**
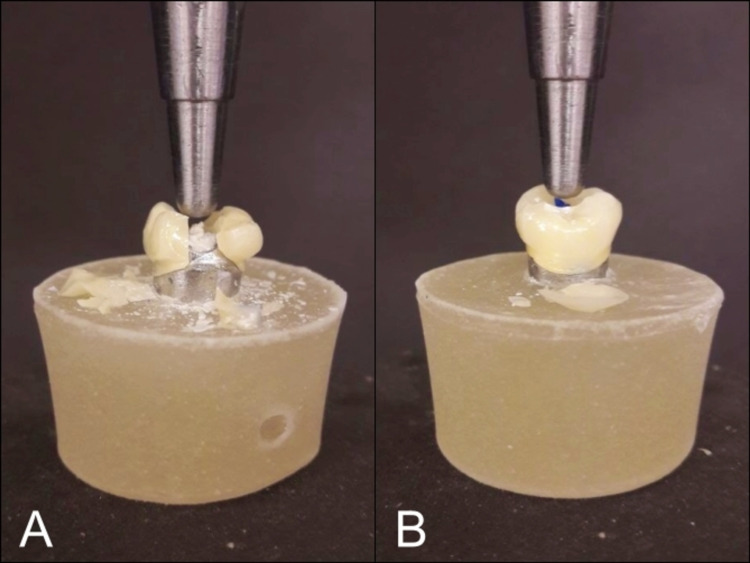
Samples of fracture mode A: core fracture; B: adhesive fracture

Two types of fracture mode were detected, either a core fracture which included both the core and the veneer, or an adhesive fracture which affected the bonding area between the veneer and the core. All crowns in the HTZ group revealed a core fracture (Figure 4A). On the other hand, six crowns in the PVZ group showed a core fracture, while four crowns showed an adhesive fracture (Figure 4B).

## Discussion

This study examined the fracture resistance of HTZ crowns and PVZ crowns. 

In this study, a CAD/CAM technique was used for the fabrication of the wax veneered layers of the PVZ group to avoid the complications and variations brought on by the traditional layered veneering technique. A similar protocol was utilized in some earlier studies [10-11].

Both PVZ and HTZ crowns showed high fracture loads, which is beyond the average bite force reported in posterior teeth (446 - 1,221 N) [17-18]. This result is comparable with a previous study that showed the superiority of zirconia-based crowns compared with other applied materials, such as lithium disilicate and nanocomposite [14]. This suggests that zirconia-based crowns have a considerable safety margin in clinical applications for the majority of patients.

Unlike the veneered zirconia crowns, which had 0.6 mm uniform thickness zirconia copings, the monolithic zirconia crowns had the thickness of full anatomic crowns. The increasing values in progressing from dental veneering zirconia to zirconia indicate an increasing shielding capacity so that higher loads are needed to deliver enough stress to the interior to propagate the internal fracture [13]. The mode of failure supports this justification. It was identified that six of 10 PVZ crowns failed in this study. This implies that the use of monolithic zirconia crowns is favored over conventional porcelain veneered copings, especially in the area subjected to an eminent biting force (such as the first molar).

Since the monolithic crowns were fabricated based on the manufacturer's instructions without a need for a porcelain veneer, as expected, the core fracture was the standard fracture mode. However, in porcelain-veneered crowns, two types of fracture modes occurred: core fractures and adhesive fractures. PVZ crowns exhibited adhesive failures in four of the samples. The reason can be attributed to the development of tensile stresses at the interior surface of the veneering layer emerging from the presence of a moderately weak intermediate layer [4].

In the current study, the null hypothesis was rejected as a significant difference in the mean fracture load existing between the PVZ and HTZ groups. The mean fracture load of the HTZ group was more than double that of the PVZ group.

In this study, some limitations should be acknowledged. As in all in vitro studies, it cannot perfectly duplicate the exact clinical situations. Therefore, the results obtained from in vitro analyses may not entirely predict the results in the normal dentition. Also, other impact factors, such as lateral and oblique forces, were not tested in this study. Also, working on metal dies does not provide the equivalent modulus of elasticity as in natural teeth which could influence the fracture resistance values.

## Conclusions

The following conclusion can be drawn within the limitations of this in vitro study: the monolithic HTZ crowns are expected to exhibit better clinical performance compared to PVZ. However, the fracture strength of both crowns types surpasses the maximum bite force in the posterior region, which may be deemed clinically adequate. More in-situ studies are needed to yield a more reliable estimation performance for clinical cases.
